# Objective and subjective measures of sleep in men with Muscular Dystrophy

**DOI:** 10.1371/journal.pone.0274970

**Published:** 2022-09-22

**Authors:** Christopher I. Morse, Gladys Onambele-Pearson, Bryn Edwards, Sze Choong Wong, Matthew F. Jacques

**Affiliations:** 1 Department of Sport and Exercise Sciences, Manchester Metropolitan University, Manchester, United Kingdom; 2 The Neuromuscular Centre, Winsford, United Kingdom; 3 Developmental Endocrinology Research Group, Royal Hospital for Children, University of Glasgow, Glasgow, United Kingdom; 4 School of Life Sciences, University of Nottingham, Nottingham, United Kingdom; National Taiwan University Hospital, TAIWAN

## Abstract

**Purpose:**

Despite poor sleep quality being recognised in Duchenne Muscular Dystrophy, reports from milder forms of Muscular Dystrophy (MD), and accompanied associations with quality of life (QoL), pain and fatigue, remain limited however.

**Methods:**

Adult males (n = 15 Beckers MD (BMD), n = 12 Limb-Girdle MD (LGMD), n = 12 Fascioscapulohumeral (FSHD), n = 14 non-MD (CTRL)) completed assessments of body composition (Bio-electrical impedance), sleep (7-day 24-hour tri-axial accelerometer, Pittsburgh Sleep Quality Index (PSQI) and Insomnia Severity Index, QoL (SF36-v2), pain (Visual analogue scale), fatigue (Modified Fatigue Index Scale) and functional assessments (Brookes and Vignos).

**Results:**

FSHD and BMD reported worse sleep than CTRL on the PSQI. FSHD scored worse than CTRL on the Insomnia Severity Index (P<0.05). 25–63% and 50–81% of adults with MD reported poor sleep quality using the Insomnia Severity Index and PSQI, respectively. Accelerometery identified no difference in sleep quality between groups. Associations were identified between sleep measures (PSQI global and insomnia severity) with mental or physical QoL in LGMD, BMD and FSHD. Multiple regression identified associations between sleep impairment and fatigue severity (all MDs), body composition (BMD & LGMD), upper and lower limb function (LGMD, FSHD) and age (FSHD).

**Conclusions:**

25–81% of men with MD, depending on classification, experience sleep impairment, using self-report sleep measures. Whilst BMD and FSHD showed worse sleep outcomes than CTRL, no group difference was observed between LGMD and CTRL, however all groups showed associations with sleep impairment and higher levels of fatigue. These findings, and associations with measures of health and wellbeing, highlight an area for further research which could impact QoL in adults with MD.

## Introduction

Muscular Dystrophy (MD) is an umbrella term for a set of progressive muscle weakness conditions, for which the focus of research and interventions has been genetics and clinical characteristics, in particular muscle strength and the maintenance of ambulation in Duchenne MD (DMD) [1–4]. More recently, an increasing volume of research has been developed in order to identify influences on quality of life (QoL) and health status in other forms of MD [5–10]. The Health Status in MD model by de Groot et al. [11] acknowledges the importance of sleep disturbances along with pain and fatigue as contributing factors to QoL and health in MD. Evidence surrounding prevalence and impact of pain and fatigue on QoL is relatively well-established [12–15], sleep disturbances on the other hand, and the broader concept of sleep quality, remains comparably under-reported in adults with MD.

The term ‘Sleep Quality’ is commonly used to encompass a variety of sleep measures, including sleep time, onset of sleep, sleep efficiency and sleep disruptions [16], with poor sleep quality a defining feature of insomnia [17]. Sleep quality is known to be reduced in conditions with chronic pain [18, 19], and has been shown to result in sensations of fatigue in other clinical conditions [18]. Both pain and fatigue have been shown to be symptomatic within MD [6, 14, 20–22], indeed, our research group recently presented associations between both pain and fatigue with QoL in adults with MD [5]. Despite the presentation of pain and fatigue in MD [5, 14], well established associations with sleep quality in other clinical conditions [19], and inclusion within the Health Status in MD model [11], direct evidence of associations between sleep quality with pain, fatigue and QoL remains limited in adults with MD, with the exception of DMD [23].

In adults with DMD, poor sleep quality is linked to a combination of supine body position, severely weakened respiratory system, and the use of a night-time ventilator (indeed, severe respiratory weakness can often result in the daytime use of a ventilator also [23–28]. In comparison, reports of sleep quality within milder conditions of MD, such as Beckers MD (BMD), Limb-Girdle MD (LGMD) and Fascioscapulohumeral MD (FSHD), are limited. In LGMD and FSHD, sleep disordered breathing has been identified previously [29–31] using recall questionnaires (LGMD and FSHD) and polysomnography (FSHD) [22]. Accelerometery as a technique has been used recently to identify impaired sleep quality in children with DMD [28], however this measurement technique has not yet been adopted in adults with milder forms of MD. While understanding of sleep quality in LGMD and FSHD is limited to these aforementioned studies [29–31], further research is required to understand the prevalence of poor sleep quality in adults with BMD, LGMD and FSHD, but also its implications on Health related QoL and associations with pain and fatigue.

Therefore this research study aimed to: 1) Assess sleep quality in adults with BMD, LGMD and FSHD using 3 methods, 2) Assess the relationship between sleep quality and QoL, 3) Identify relationships between Pain, Fatigue and Participant Characteristics, and sleep quality.

## Material and methods

Fifty-Three adult males volunteered to participate in this study (n = 15 BMD, n = 12 LGMD, n = 12 FSHD, n = 14 non-MD controls (CTRL), Table 1). Participants were grouped by dystrophic condition which has been confirmed by gene analysis in their referral. All participants with MD were recruited from, and tested at, The Neuromuscular Centre (Winsford, UK), where they habitually participate in monthly physiotherapy sessions (Average monthly attendance = 2 ± 0.34 days). CTRL participants, were recruited from the general population and free from any health conditions. CTRL participants were tested at the local university campus, using identical methods and equipment as the MD participants (with the exception of height and body mass, see below). None of the MD or CTRL participants reported any change in their activity levels or physiotherapy provision in the 3-months prior to inclusion in this study. All participants arrived for their testing in a fasted state. Ethical approval was obtained through the Sports and Exercise Science Ethics Committee, and all participants provided informed written consent prior to participation. All procedures complied with the World Medical Association Declaration of Helsinki [32].

**Table 1:** Participant characteristics and body composition.

	BMD	LGMD	FSHD	CTRL
**N**	15	12	11	14
**Age (Years)**	40.7 (12.3)	43.3 (11.3)	46.3 (12.4)	37.8 (12.8)
**Mass (Kg)**	79.5 (13.1)	97.6 (17.3)	87.2 (12.1)	84.2 (17.9)
**Stature (cm)**	178.3 (5.9)	179.6 (7.2)	180.2 (8.3)	179.5 (8.0)
**BMI**	25.1 (3.5)	29.4 (4.5)	27.2 (3.7)	26.0 (4.0)
**Body Fat (%)**	26.1 (7.6)^C^	33.8 (4.8)^F^^,^^C^^,^^B^	26.4 (7.8)^C^	18.4 (5.0)
**Lean Body Mass (Kg)**	58.2 (8.5) ^C^	64.4 (8.7)	62.6 (8.9)	68.4 (12.7)
**Brooks**	2 (1–3)	2 (2–4)	2 (1–3)	-
**Vignos**	6 (2–9)^F^	9 (3–9)^F^	3 (1–9)	-
**Respiratory Support**				
*Night Time Only*	0/15	2/12	0/11	-
*24–7*	0/15	0/12	0/11	-

Participant Characteristics. Data presented as Mean (SD), or median (range) where appropriate. Significant differences are denoted in those furthest away from CTRL first

^C^ denotes significantly different from CTRL

^B^ denotes significantly different from BMD

^LG^ denotes significantly different for LGMD

^F^ denotes significantly different from FSHD. Kg = Kilograms; cm = centimetres; BMI = body mass index; BMD = Beckers Muscular Dystrophy; LGMD = Limb-Girdle Muscular Dystrophy; FSHD = Fascioscapulohumeral Muscular Dystrophy; CTRL = Control.

### Procedures

Each participant was tested in a single testing session. The same equipment was used for all participants, with the exception of seated scales for body mass measures in non-ambulatory MD participants. Anthropometric measures were performed first, followed by questionnaires for sleep quality, insomnia, quality of life, fatigue and pain were completed independently. The principal investigator was present to aid with any questions, or in some cases, to tick the desired box for participants with limited upper-limb function. Upon completion of questionnaires, an accelerometer was strapped to the wrist of the self-reported dominant arm and worn for seven consecutive days and nights (GENEActiv, Cambridge, United Kingdom).

### Anthropometry

Control participants’ mass was measured whilst standing (unshod) using digital scales (Seca model 873, Seca, Germany). MD participants were weighed in digital seated scale (6875, Detecto, Webb City, Mo, USA). Slings, shoes, splints etc. were weighed separately and subtracted from the gross weight. All participants’ stature was calculated as point to point (index finger, elbow, shoulder and across midline) to replicate a previously used method on non-ambulatory participants [33, 34]. A correction of 3.5% was applied to the raw data, consistent with regression data from Caucasian males in order to account for the known discrepancy between height and arm span measures [35].

Body Mass Index (BMI) was calculated using the following equation [36]:

$$BMI\;(\frac{Kg}{m^{2}})=Body\;Mass\;(Kg)÷Height^{2}\;(m^{2})$$

### Body composition

Body composition measures of lean body mass (LBM) and fat mass (FM) were measured using BIA in a fasted state. Two adhesive electrodes were placed on the dorsal surfaces of the metacarpals and metatarsals of the right foot and hand. Two proximal electrodes were placed between the medial and lateral malleoli of the right ankle, and between the medial and lateral malleoli of the right radius and right ulna. BIA is a promoted method of body composition assessment in MD, given its speed and ease of use within populations that may be non-ambulant. BIA has been promoted as a measure for change in FM and LBM over time in MD [37].

### Functional scales

Lower and upper limb function was assessed using common functional scales in MD, namely Brooke [38, 39] and Vignos [1] scales, respectively. The Brooke scale ranges from 1–6, whereby 1 means the participant is able to “start with arms at the sides and abduct the arms in a full circle until they tough above the head” and 6 means “Cannot raise hands to the mouth and has no function of hands” [40]. The Vignos scale ranges from 1–10, with 1 “Walk and climb stairs without assistance, and 10 “Confined to a bed” [40]. All functional scales were performed by a chartered physiotherapist, and are reported in MD participants only [14, 41].

### Sleep assessment

#### Pittsburgh sleep quality index

The Pittsburgh Sleep Quality Index (PSQI) is a reliable (α = 0.73–0.81) [42–44] and commonly used measure of sleep quality within clinical and research settings [23, 45, 46]. The PSQI is a 21-item questionnaire, determining a global score, representing the sum of its seven domain scores: Subjective Sleep Quality, Sleep Latency, Sleep Duration, Habitual Sleep Efficiency, Sleep Disturbances, Use of Medications for Sleep and Daytime Dysfunction. Individual domains are scored on a scale of 0–3, whereby 0 denotes no difficulty, and 3 denotes severe difficulty. “Poor sleep quality” is determined from a global score of 5 or greater.

#### Insomnia severity index

The Insomnia Severity Index is a self-report measure of Sleep that has been widely used in clinical conditions [47–50], consisting of 7 items. These 7 items are: Severity of sleep-onset, Sleep Maintenance, Early morning wakening, Satisfaction with sleep pattern, interference with daily functioning, impairment attributed to sleep and distress caused by sleep [51]. Questions are on a 5-point Likert scale, from “Not at all (0) to “extremely” (4), Total scored range from 0–28, with higher scores indicative of greater insomnia severity. Total scores up to 7 is considered ‘Not Clinically Significant Insomnia’, scores 8–14 are considered ‘Subthreshold Insomnia’, Scores 15–21 are considered ‘Clinical Insomnia (Moderate)’, and scores 22–28 are considered ‘Clinical Insomnia (Severe) [52].

#### Accelerometer

Participants wore a wrist-watch triaxial accelerometer (GENEACTIV, Kimbolton, Cambs, United Kingdom) over a consecutive 7 day period [53], which has been reported as reliable and valid previously [54, 55]. All participants wore accelerometers on the wrist to increase adherence and remove the potential discomfort of waist-worn accelerometers for non-ambulant participants [56]. Furthermore, the use of accelerometers to assess sleep has shown 83–89% agreement with polysomnography [57, 58], and has become a more common assessment method due to participant convenience compared to polysomnography [59–62]. Wrist-worn accelerometers were worn for 24h a day on the self-reported dominant wrist of the participant, with daily physical activity reported previously by the authors [63]. Monitors were initialised to collect data at 100Hz. Once the monitors were returned, post 7-day data collection, data was downloaded from monitors into.bin files and converted in 60s epoch.csv files using the GENEACTIV PC Software (Version 2.1). 60s epoch data files were entered into a validated open source Excel Macro (v2, Activinsights Ltd.) [64]. The Excel macro reports participant bed time, rise time, total elapsed sleep time (time between bed time and rise time), sleep time (time actually asleep), Sleep Efficiency (Sleep Time/ Total Elapsed Sleep time), number of activity periods and median activity period length. This paper presents data on average Sleep Time, Sleep Efficiency, Number of Activity Periods and Activity Period Length for analysis.

#### Pain

A Visual Analog Scale of pain (Pain VAS) was used to quantify the level of whole body pain felt by participants over the 7 days preceding assessment. VAS is a common method of pain assessment [65] and used in many conditions [66, 67]. Participants were given a 10cm straight line, with at one end “No Pain”, and the other “Worst Possible Pain”, and instructed to mark where, on average, they felt their pain over the preceding 7 days was on the scale. The mark was then measured and presented as distance (cm) from the “No Pain” end.

#### Fatigue

The Modified Fatigue Index Scale is a 21-item questionnaire, was completed by participants to provide a total fatigue score (MFIS Total) or a subscore of its domains, namely Physical (MFIS Physical), Cognitive (MFIS Cognitive) or Psychosocial (MFIS Psychosocial). Participants rate the 21 questions on a 5-point Likert scale, from “Never” (0) to “Always” (4). MFIS Total is out of a possible score of 84, with scores 38 or over indicative of Fatigue.

#### Quality of life

All participants completed the SF-36v2 questionnaire, a reliable and validated measure, with eight domains of quality of life (QoL) [68, 69], for which full description is available in our research group’s previous work [5]. For the purpose of the present paper, data is presented as composite Total Mental (QoL Mental) and Total Physical (QoL Physical) component scores. All data was analysed using Health Outcomes Scoring Software 4.5 (QualityMetric Health Outcomes™, Lincoln, United Kingdom).

### Statistical analysis

All analyses were performed using IBM Statistics 26 software. The critical level of statistical significance was set at 5%. All data, except for stature and LBM, were non-parametric as determined through Levene’s and Shapiro-Wilk tests. Kruskal-Wallis test was used to compare between groups with post hoc Mann-Whitney U (least significant difference) pairwise comparisons used where appropriate. Stature and LBM were compared between groups using a one-way analysis of variance (ANOVA), with Tukey’s test used for post hoc pairwise comparisons. Where appropriate, bias corrected accelerated confidence intervals were calculated with 1000 bootstrap replicates [70]. Linear regressions between Sleep Quality (PSQI—Global Score; Insomnia Severity Index—Total Score; and Accelerometer—Sleep Time and Sleep Efficiency) and QoL Measures (SF36-v2 Physical and SF36-v2 Mental) were conducted. Stepwise Multiple Linear Regressions were conducted between co-variates (Pain, Fatigue and Participant Characteristics of Age, Fat Mass, Fat Free Mass, Vignos and Brookes rating) and measures of Sleep Quality (PSQI—Global Score; Insomnia Severity Index—Total Score; and Accelerometer—Sleep Time and Sleep Efficiency). ANCOVA was conducted to determine whether there were group differences in QoL (Physical and Mental) while controlling for covariables of fatigue, pain, and sleep (PSQI global) with Bonferroni post-hoc. Significant differences in tables and figures are denoted in the MD group furthest from CTRL in comparison, and thereafter from most affected to least affected MD.

## Results

### Participant characteristics

No differences were identified between groups for Age, Stature, Mass or BMI (P>0.05, Table 1). BMD (42%, P = 0.10, 95% CI [1.5, 14.1]), LGMD (84%, P<0.001, 95% CI [8.9, 22.2]) and FSHD (44%, P = 0.036, 95% CI [0.4, 14.4]) had higher body fat % than CTRL (Table 1). In addition LGMD had higher body fat % than BMD (30%, P = 0.015, 95% CI [1.2, 14.3]) and FSHD (28%, P = 0.022, 95% CI [-15.4, -0.9]). LBM was 15% lower in BMD compared to CTRL (P = 0.04, 95% CI [-20.0, -0.5], Table 1). There were no other anthropometric differences (P>0.05).

### Functional scales

FSHD participants scored lower on the Vignos Scale than BMD (33%, P = 0.044, Table 1) and LGMD (67%, P = 0.002, Table 1), while no differences were identified between BMD and LGMD participants. No differences were identified between MD groups for Brooks Scale (P>0.05, Table 1).

### Sleep

For clarification, higher domain and global scores for PSQI, as well as Insomnia Severity Index, are indicative of worse measures of sleep.

**Fig 1:**
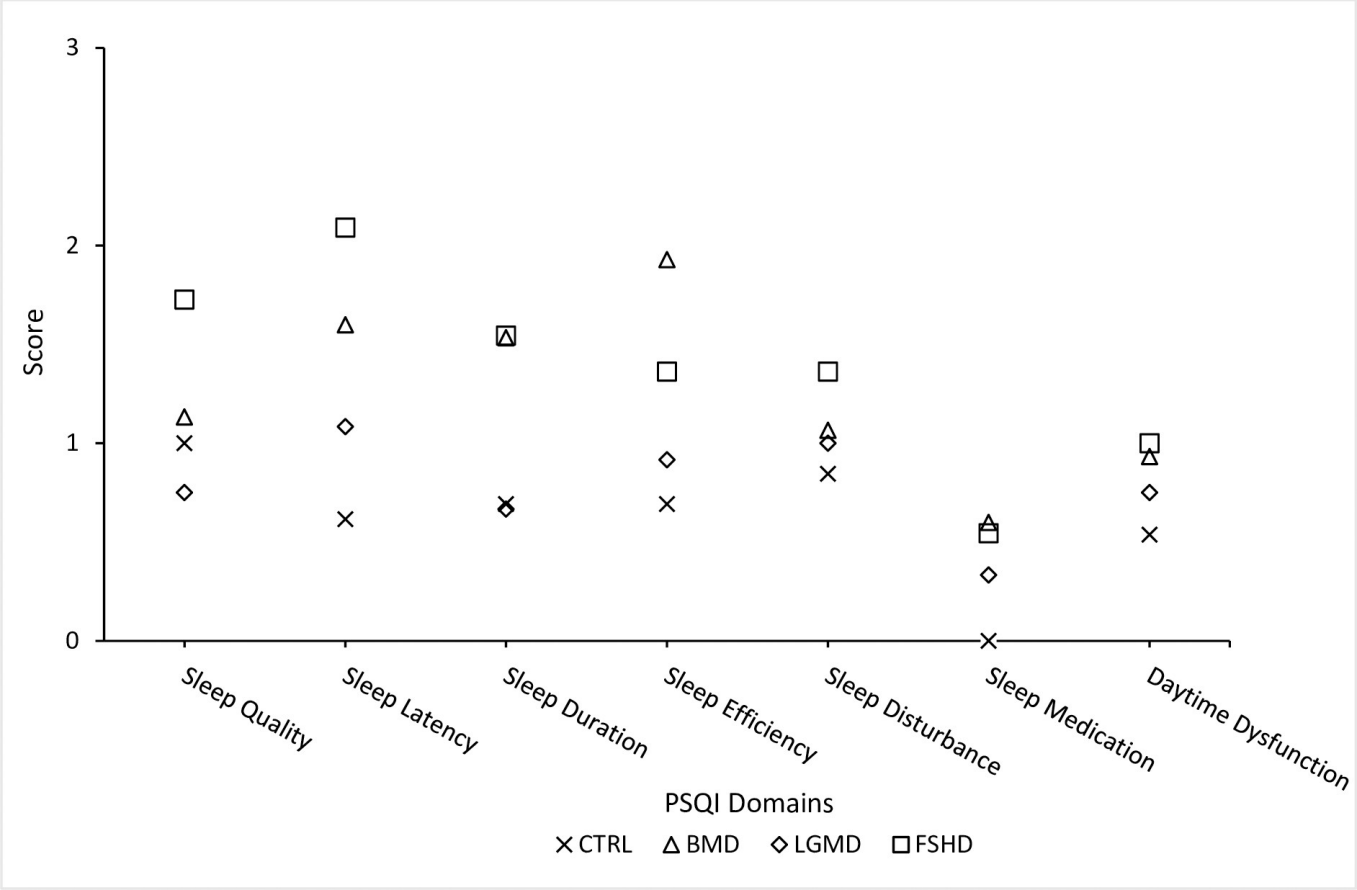
Group scores for PSQI domains. For clarity, differences between groups are described in text but not displayed on this Figure.

FSHD participants scored higher in the PSQI Sleep Quality Domain than LGMD (130%, P = 0.02, 95% CI [0.12, 1.81], Fig 1). FSHD participants scored higher in the PSQI Sleep Latency Domain than CTRL (240%, P = 0.006, 95% CI [0.35, 2.62], Fig 1). BMD participants scored higher in the PSQI Sleep Efficiency Domain than CTRL (88%, P = 0.008, 95% CI [2.18, 2.52], Fig 1). No other differences were identified between groups for PSQI Domains (P>.05), however all data are presented in Fig 1.

FSHD and BMD participants scored higher on the PSQI Global Score than CTRL (88%, P = 0.011, 95% CI [0.96, 9.50] and 123%, P = 0.032, 95% CI [0.26, 7.93], respectively, Table 2). No other difference were identified between groups.

**Table 2:** Sleep measures in adults with MD.

	BMD	LGMD	FSHD	CTRL
**PSQI**				
Global Score	8.7 (4.7)^C^	5.5 (3.3)	10.3 (5.0)^C^^,^^L^	4.6 (2.0)
Prevalence of Poor Sleep Quality	80%	50%	81%	43%
**Insomnia Severity Index**				
Total Score	8.40 (6.1)	5.25 (5.6)	11.0 (6.7)^C^^,^^L^	3.85 (2.9)
No Clinically Significant Insomnia	40%	75%	36%	86%
Subthreshold Insomnia	40%	17%	36%	14%
Moderate Severity Insomnia	20%	8%	27%	-
Severe Insomnia	-	-	-	-
**Accelerometer**				
Sleep Time (mins)	411 (142)	426 (146)	428 (86)	392 (62)
Sleep Efficiency (%)	67% (15%)	70% (17%)	70% (10%)	77% (12%)
Activity Periods (n)	12.2 (5.0)	10.6 (5.6)	10.6 (5.4)	10.5 (3.6)
Activity Period (mins)	10.8 (3.0)	13.4 (11.0)	13.1 (8.8)	8.6 (2.9)

Measures of Sleep in Adults with MD. Data presented as Mean (SD). Significant differences are denoted in those furthest away from CTRL first

^C^ denotes significantly different from CTRL

^B^ denotes significantly different from BMD

^LG^ denotes significantly different for LGMD

^F^ denotes significantly different from FSHD. mins = minutes; PSQI = Pittsburgh Sleep Quality Index; BMD = Beckers Muscular Dystrophy; LGMD = Limb-Girdle Muscular Dystrophy; FSHD = Fascioscapulohumeral Muscular Dystrophy; CTRL = Control.

FSHD participants scored higher on the Insomnia Severity Index Total Score than CTRL participants (186%, P = 0.015, 95% CI [1.06, 13.2], Table 2). No other differences were identified between groups for Insomnia Severity Index Total Score (P>0.05, Table 2).

No differences were found between groups for any of the accelerometer determined measures of sleep time, sleep efficiency, number of activity periods or activity period length (P>0.05).

### Pain, fatigue and quality of life

Pain rating was higher in BMD (P = 0.002, 95% CI 0.92, 5.22), LGMD (P = 0.004, 95% CI [0.84, 5.39]) and FSHD (P<0.001, 95% CI [1.18, 5.97]) compared to CTRL (Table 3). Physical fatigue (MFIS Physical) was higher in BMD (P<0.001, 95% CI [8.9, 21.0]), LGMD (P<0.001, 95% CI [10.1, 22.9]) and FSHD (P<0.001, 95% CI [-30.4, -16.9]), compared to CTRL (Table 3). BMD participants had lower physical fatigue than FSHD (P = 0.006, 95% CI [-15.4, -2.1], Table 3). Cognitive fatigue (MFIS cognitive) was higher in FSHD than CTRL (P = 0.03, 95% CI [0.6, 15.4]. Psychosocial fatigue (MFIS Psychosocial) was higher in BMD (P<0.001, 95% CI [1.74, 4.84]), LGMD (P = 0.001, 95% CI [1.30, 4.58]) and FSHD (P<0.001, 95% CI [3.33, 6.78]) than CTRL (Table 3). LGMD participants had lower psychosocial fatigue than FSHD (P = 0.014, 95% CI [-3.90, -0.33], Table 3). Overall fatigue (MFIS total) was higher in BMD (P<001, 95% CI [11.3, 37.3]), LGMD (P = 0.001, 95% CI [9.33, 36.8]) and FSHD (P<0.001, 95% CI [22.2, 51.2]) compared to CTRL (Table 3). SF36-v2 Physical was lower in BMD (27%, P<0.001, 95% CI [-27.6, -16.1]), LGMD (33%, P<0.001, 95% CI [-32.3, -20.1]) and FSHD (32%, P<0.001, 95% CI [-31.9, -19.1]) participants compared to CTRL (Table 3). No other differences were identified between groups (P>0.05). ANCOVA revealed group differences in QoL (Physical) (P<0.001) when controlling for pain, fatigue (MFIS Total), and sleep (PSQI global), between all MD groups and CTRL: BMD (P<0.001, 95% CI [-21.7, -9.2]), LGMD (P<0.001, 95% CI [-27.6, -14.1]) and FSHD (P<0.001, 95% CI [-23.1, -8.0]). There was no group or covariance effect between QoL (Mental) and pain, fatigue, and sleep.

**Table 3:** Pain, fatigue and quality of life in adults with MD.

	BMD	LGMD	FSHD	CTRL
**Pain**				
VAS Pain	3.50 (2.6)^C^	3.54 (2.7)^C^	3.73 (2.2)^C^	0.43 (0.8)
**Fatigue**				
MFIS Physical	19.6 (7.4)^C^	21.2 (6.6)^C^	27.9 (4.4)^C^^,^^B^	4.64 (5.1)
MFIS Cognitive	11.3 (8.1)	8.83 (6.0)	12.5 (6.9) ^C^	5.21 (5.4)
MFIS Psychosocial	3.93 (2.0)^C^	3.58 (1.9)^F^^,^^C^	5.6 (1.1)^C^	0.64 (0.9)
MFIS Total	34.8 (16.6)^C^	33.6 (12.2)^C^	45.9 (11.3)^C^	10.5 (10.8)
**Quality of Life**				
SF36-v2 Physical Score	34.7 (7.3)^C^	30.4 (5.4)^C^	32.0 (6.5)^C^	56.3 (3.8)
SF36-v2 Mental Score	51.0 (8.6)	57.1 (9.0)	49.5 (10.9)	56.2 (3.8)

Pain, Fatigue and Quality of Life in Adults with MD. Data presented as Mean (SD). Significant differences are denoted in those furthest away from CTRL first

^C^ denotes significantly different from CTRL

^B^ denotes significantly different from BMD

^LG^ denotes significantly different for LGMD

^F^ denotes significantly different from FSHD. MFIS = Modified Fatigue Index Scale; VAS = Visual Analogue Scale; BMD = Beckers Muscular Dystrophy; LGMD = Limb-Girdle Muscular Dystrophy; FSHD = Fascioscapulohumeral Muscular Dystrophy; CTRL = Control.

### Associations with sleep measures

#### QoL

Linear regression identified significant relationships for PSQI with QoL Mental in LGMD (R^2^ = 0.593, P = 0.003, Table 4), and QoL Physical in BMD (R^2^ = 0.302, P = 0.034, Table 4). Linear regression identified significant relationships for Insomnia Severity with QoL Mental in LGMD (R^2^ = 0.371, P = 0.036, Table 4), and Physical QoL in FSHD (R^2^ = 0.382, P = 0.043, Table 4). No other significant relationships were identified between QoL and Sleep Quality (P>0.05).

**Table 4:** Associations between sleep measures and QoL.

	PSQI	Insomnia Severity Index	Accelerometer	
	*Global Score*	*Total Score*	*Sleep Time*	*Sleep Efficiency*
SF36-v2 Physical	0.302^B^	0.382^F^	-	-
SF36-v2 Mental	0.593^LG^	0.371^LG^	-	-

Associations between sleep measures and QoL measures. Data presented as R values between corresponding variables.

^B^ denotes significant association for BMD participants

^LG^ denotes significant association for LGMD participants

^F^ denotes significant association for FSHD participants. MFIS = Modified Fatigue Index Scale; VAS = Visual Analog Scale; BMI = Body Mass Index.

#### Multiple linear regressions

In BMD, MFIS Physical was identified as the strongest relationship with PSQI (R^2^ = 0.588, P = 0.001), with all other variables excluded. Body Fat (%) was identified as the strongest relationship with Severity Index (R^2^ = 0.358, P = 0.018), with all other variables excluded. No models were identified including any variables for Sleep Time or Sleep Efficiency in BMD (P>0.05).

In LGMD, Brooke, Vignos, Body Fat (%) and Age were identified as the strongest relationship with Sleep Efficiency (R^2^ = 0.952, P<0.001), with all other variables excluded. MFIS Total was identified as the strongest relationship with PSQI (R^2^ = 0.332, P = 0.049), while MFIS Cognitive was identified as the strongest relationship with Insomnia Severity Index (R^2^ = 0.548, P = 0.006), with all other variables excluded. No model was identified including any variables however for Sleep Time in LGMD (P>0.05).

In FSHD, Vignos and MFIS Physical were identified as the strongest relationship with Insomnia Severity Index (R^2^ = 0.770, P = 0.003), with all other variables excluded. MFIS Physical was identified as the strongest relationship with PSQI (R^2^ = 0.408, P = 0.034), with all other variables excluded. Age and MFIS Cognitive were identified as the strongest relationship with Sleep Efficiency (R^2^ = 0.652, P = 0.015). No model was identified including any variables however for Sleep Time in FSHD (P>0.05).

## Discussion

This study has assessed sleep in adults with BMD, LGMD and FSHD using three separate assessments. Between 25–81% of Adults with MD, depending on classification, experience some form of sleep impairment compared to 14–43% of CTRL, using self-report measures of sleep impairment. The severity of sleep impairment in adults with MD was associated with low physical function, higher body fat, higher levels of fatigue and lower QoL. These findings, although consistent with work in children with DMD [23, 26–28], could represent a new avenue for potentially improving QoL in adults with MD.

While using accelerometery, adults with MD showed comparable sleep to CTRL, in contrast, self-reported outcomes consistently identified impaired sleep quality in Adults with MD. Of particular note, using the PSQI, poor sleep quality was noted in 80% and 81% of adults with BMD and FSHD, respectively. In comparison, Della Marca, Frusciante (30) reported 59% of adults with FSHD had poor sleep quality using the same assessment, while more recently Crescimanno et al. [23] reported 56% adults with DMD using night time ventilators had poor sleep quality, with a PSQI Global Score of 6.1, within the present study PSQI Global Score ranged from 5.5 (LGMD) to 10.3 (FSHD). The increased prevalence of impaired sleep quality in the present study, despite participants not using night-time ventilators may be surprising. It is possible that physical limitations and an impaired ability to re-position during sleep may explain these findings, given that accelerometers did not identify differences in sleep quality. This is further evidenced by the associations identified between physical function, namely the Brooks (FSHD) and Vignos (LGMD, FSHD) scales, and either the Insomnia Severity Index or sleep efficiency. It is pertinent to note, that despite being a valid measure of sleep compared with polysomnography [57, 58], there is a lack of polysomnography data from adults with MD from which we can make a comparison. Regardless, in the present study, adults with MD had a 50–81% prevalence of poor sleep quality (PSQI), and 8–27% had moderate insomnia (Insomnia Severity Index), emphasising the need for further investigation into impaired sleep quality in adults with MD.

**Fig 2:**
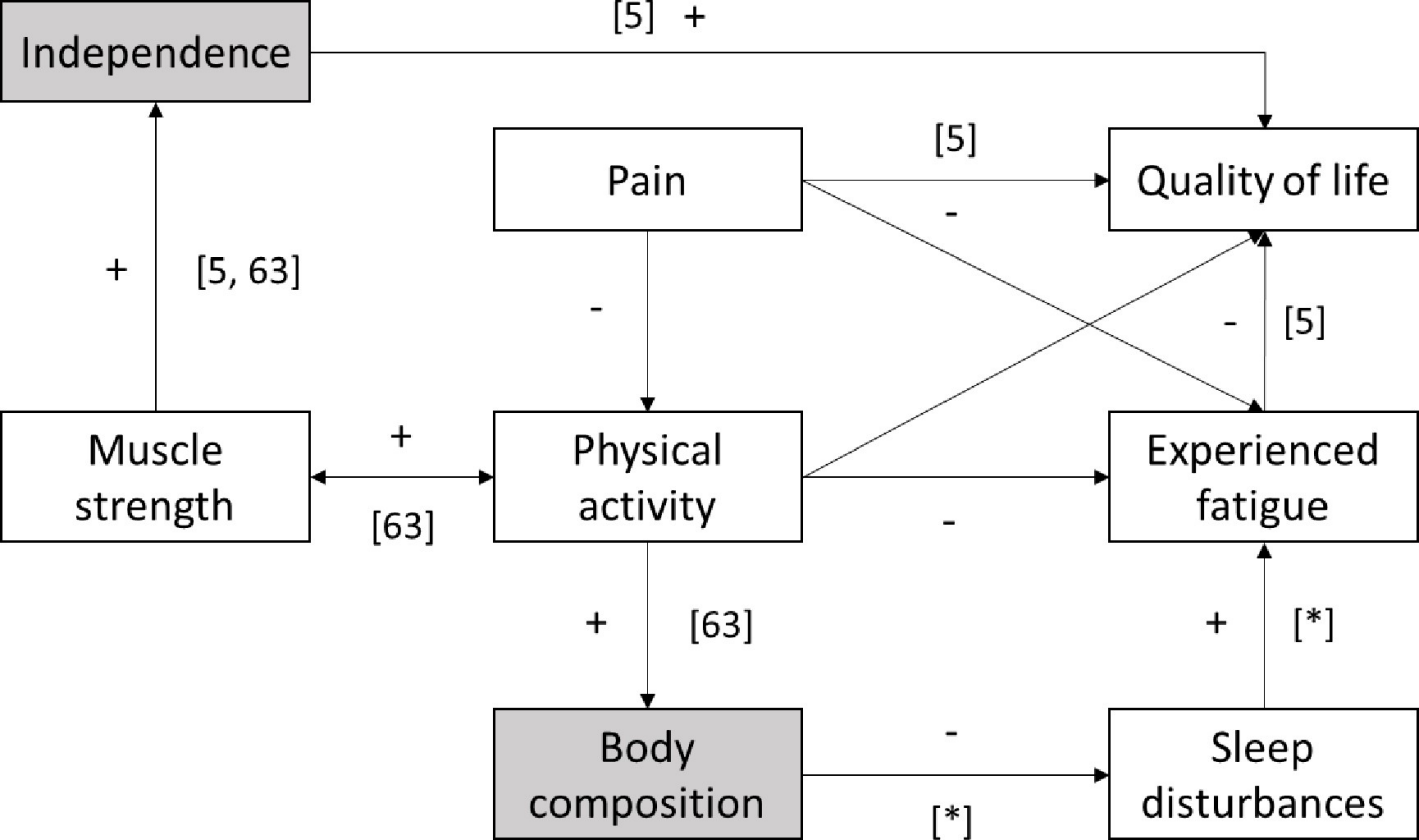
Proposed health model for adults with muscular dystrophy, adapting the previous work from de Groot et al. [11] (White Boxes), added new components (Grey Boxes), using the present study (Denoted by [*]) and the authors previous findings [5, 63]. Increases/improvements or decreases/impairments are denoted by + or–signs, respectively.

Despite experiencing low QoL, high pain and high fatigue, those with LGMD did not present differences in sleep outcomes from CTRL, despite having a 50% prevalence of participants with poor sleep quality. Of interest however was the within group associations, showing that those with poor sleep tended to have higher body fat consistent with BMD, and higher levels of fatigue (MFIS total), consistent with BMD and FSHD. The present study has therefore, provided further evidence for the health model proposed previously by de Groot et al. [11], through associations between sleep in multiple classifications of MD and QoL. In addition, associations were consistently identified between measures of body composition, function scales and fatigue with Sleep Measures in adults with MD in the present study. Previous work has reported associations between sleep disruption and respiratory impairment in LGMD [29], these respiratory impairments are likely exacerbated by body fat% and BMI associations identified in the present study, resulting in apnoea and a likely eventual need for nocturnal ventilation [30, 31]. While relationships between visceral fat and sleep apnoea are recognised within healthy populations [71], further research is required to explore the relationships between absolute adiposity and fat distribution with sleep in adults with MD [72]. Associations between sleep impairment and functional measures, fatigue and QoL in the present study are similar to those reported previously in FSHD [22]. Fatigue has become a prevalent feature of MD research [13, 73], particularly within FSHD [21, 74], whereby the current authors reported its impact on QoL across adults with MD previously [5]. Therefore, the present authors propose an updated health model for adults with MD (Fig 2), whereby independence, body composition and sleep disturbances are included and evidenced to impact QoL across adults with MD, through our current and previous work [5, 63], as well as work evidenced in the original de Groot model [11].

Using the updated health model, it could be suggested that physical activity may be a key area to improve QoL and health status in adults with MD. The effect of increased physical activity on improvements in body composition and reducing body fat is well established within the general population [75] and recognised in MD [33, 63, 76, 77]. This may therefore reduce the effect of increased body fat percentage on sleep disturbances in adults with MD. Furthermore, Voet et al. [74] has reported previously how increased aerobic exercise resulted in decreased experienced fatigue in adults with FSHD, however the applicability across MD conditions remains unexplored. The description of Physical behaviour (during waking hours from physical activity through to sedentary behaviours) is relatively limited in adults with MD however [63, 78], future studies should consider the potential benefits of physical activity interventions on fatigue, sleep quality and QoL in adults with MD.

### Limitations

It should be recognised that the MD population recruited for this study voluntarily attend a centre for care and rehabilitative treatments. Participants must be referred to the centre via a clinician, therefore participants may be pre-disposed to higher levels of physical dysfunction, pain or poor sleep quality, resulting in their referral. In addition, this study has used a relatively novel method of accelerometery to assess sleep quality in a clinical population [28]. Accelerometers allow for an objective method to be used within a home setting, rather than the intrusive nature of polysomnography [79]. Outcome measures however are estimates of sleep quality based on movement, and as our results attest, such should be treated with some caution. The present study included two self-report methods of sleep quality to supplement the objective method of assessment, and consistent in previous work in children with DMD [61], all three found associations relative to the conclusions proposed.

## Conclusion

In conclusion, this study has identified a high prevalence of impaired self-reported sleep in adults with MD, along with a wide range of associations including fatigue and QoL. These findings have helped further develop an initial health model for adults with MD by de Groot et al. [11], resulting in a more comprehensive health model suggested by the present authors. This health models proposes future interventions that can improve sleep quality, will have benefits on QoL in adults with MD, and reduce some of the burden associated with these conditions.
